# A novel flow cytometry-based cell capture platform for the detection, capture and molecular characterization of rare tumor cells in blood

**DOI:** 10.1186/1479-5876-12-143

**Published:** 2014-05-23

**Authors:** Masaru Watanabe, Masakuni Serizawa, Takeshi Sawada, Kazuo Takeda, Toshiaki Takahashi, Nobuyuki Yamamoto, Fumiaki Koizumi, Yasuhiro Koh

**Affiliations:** 1 Drug Discovery and Development Division, Shizuoka Cancer Center Research Institute, Shizuoka, Japan; 2 Third Department of Internal Medicine, Wakayama Medical University, Wakayama, Japan; 3 Shien-Lab, National Cancer Center Hospital, Tokyo, Japan; 4 On-Chip Biotechnologies Co., Ltd., Tokyo, Japan; 5 Division of Thoracic Oncology, Shizuoka Cancer Center Hospital, Shizuoka, Japan

**Keywords:** Circulating tumor cells, Cell sorter, Flow cytometry, Liquid biopsy, EpCAM-independent, Next-generation sequencing, Mutation detection, Single cell analysis, Whole genome amplification

## Abstract

**Background:**

Personalized cancer treatment relies on the accurate detection of actionable genomic aberrations in tumor cells. Circulating tumor cells (CTCs) could provide an alternative genetic resource for diagnosis; however, the technical difficulties in isolating and analyzing rare CTCs have limited progress to date. In this preclinical study, we aimed to develop an improved capture system for molecular characterization of CTCs based on a novel cell sorting technology.

**Methods:**

We developed a cell capture platform using On-chip Sort (On-Chip Biotechnologies), a novel bench-top cell sorter equipped with a disposable microfluidic chip. Spike-in experiments comprising a series of lung cancer cell lines with varying epithelial cell adhesion molecule (EpCAM) expression levels were conducted to assess the capture and purification efficiency of the platform. Samples were negatively enriched using anti-CD45-coated magnetic beads to remove white blood cells, followed by sample fixation and labeling. The enriched and labeled samples were then sorted by On-chip Sort based on cytokeratin, vimentin, and CD45 expression. Captured cells were immediately subjected to whole genome amplification followed by mutation analysis using deep targeted sequencing, and copy number analysis using quantitative polymerase chain reaction (qPCR).

**Results:**

Spike-in experiments revealed an excellent overall mean capture rate of 70.9%. A 100% success rate in the detection of *EGFR*, *KRAS* and *BRAF* mutations from captured cells was achieved using pyrosequencing and deep sequencing. The mutant variant detection rates were markedly higher than those obtained with the CellSearch profile kit. qPCR analysis of amplified DNA demonstrated reproducible detection of copy number changes of the *EGFR* in captured tumor cells.

**Conclusions:**

Using a novel cell sorter, we established an efficient and convenient platform for the capture of CTCs. Results of a proof-of-principle preclinical study indicated that this platform has potential for the molecular characterization of captured CTCs from patients.

## Background

Recent advances in molecularly targeted cancer therapy have offered up a wide variety of therapeutic strategies. The presence or absence of various actionable genomic aberrations has been shown to predict response to molecularly targeted treatments [1]. In some cases, identification of genetic aberrations is a prerequisite for commencing treatment; for example, identification of *EGFR*-activating mutations in patients with non-small cell lung cancer is required prior to starting treatments with EGFR tyrosine kinase inhibitors [2]. However, kinase inhibition frequently leads to the appearance of drug resistance mutations within the target kinase itself, such as the EGFR T790M mutation [3].

In addition to identifying gene mutations, there is also a need for detection of protein expression and gene amplification of targeted molecules on primary tumor cells for further stratification of patients [4]. To optimize treatment, real-time monitoring of tumors over the course of the treatment, especially at the point of treatment failure, is necessary. However, rebiopsy remains challenging, mainly because of the invasiveness of the procedure.

Circulating tumor cells (CTCs) could potentially serve as an alternative to tumor tissue as a source of material for the detection of genetic alterations, an approach that is termed “liquid biopsy” [5-11] owing to its minimal invasiveness. To date, the CellSearch system (Veridex LLC, Raritan, NJ, USA) is the only United States Food and Drug Administration-approved CTC enumeration system for the provision of prognostic information regarding survival [12-17]. However, the isolation of the rare CTCs for molecular analysis remains technically challenging. Most of the currently available capture methods retain a considerable number of white blood cells (WBCs) and cell loss during sample handling. Various methods to overcome this issue have been under development and evaluation [18-28].

The conventional cell sorting device is a well-established cell capture system and it has previously been used to enrich CTCs from whole blood [29]. However, it is reportedly difficult to efficiently carry out this isolation when using blood samples with a low CTC count together with a conventional fluorescence-activated cell sorter [20].

Recently, we have established a protocol for CTCs enumeration using a newly-developed flow cytometry FISHMAN-R [30]. The results of preclinical study showed superior sensitivity of their system in detecting EpCAM-negative tumor cells in direct comparison with the standard method. This protocol also enables a detection of EpCAM-/CK- cells and epithelial-mesenchymal transition (EMT)-induced tumor cells using the incorporation of an EMT marker [30]. The system and protocol have been evaluated and validated for the enumeration of CTCs in clinical feasibility study [31,32].

In this study, we introduced a new approach for the characterization of CTCs captured by On-chip Sort cell sorter. This novel cell sorter is an integrated sorting unit with FISHMAN-R, allowing the detection and isolation of rare tumor cells for subsequent molecular analyses. Here we evaluate the feasibility of mutation analysis of the isolated rare cell in blood after immunomagnetic enrichment and fluorescence-activated cell sorting. This is an efficient and convenient platform based on a cell sorting system, and promising preclinical results were obtained for possible future clinical application.

## Methods

### Cell lines and culture

The tumor cell lines A431, A549, H292, HCC827, H1975, and H1755 were obtained from the American Type Culture Collection (ATCC; Lockville, MD). The breast cancer cell line Hs578T was kindly gifted by Dr. Tohru Mochizuki (Shizuoka Cancer Center Research Institute, Japan). A549, H292, HCC827, H1975, and H1755 cells were cultured in RPMI-1640 (Invitrogen, Carlsbad, CA) containing 10% fetal bovine serum (Gibco, Life Technologies, Grand Island, NY). A431 and Hs578T cells was cultured in Dulbecco’s modified Eagle’s medium (DMEM; Invitrogen) containing 10% FBS. Cell lines were cultured under humidified 5% CO_2_/95% air at 37°C.

### Blood spiking experiments

Blood samples of 4 mL each were spiked with 5–25 cells of the above-mentioned cell lines and were used for the isolation of tumor cells for mutation and gene copy number analysis. Blood samples were collected from healthy volunteers working at Shizuoka Cancer Center who consented to donation. This study was approved by the independent institutional review board of Shizuoka Cancer Center.

Tumor cells were harvested by incubation with 0.25% trypsin/EDTA (Gibco) solution for several minutes at 37°C, and then washed and resuspended in T-buffer {0.5% bovine serum albumin (Nacalai Tesque Inc., Kyoto, Japan) and 2 mM ethylenediaminetetraacetic acid (EDTA; Sigma-Aldrich, St. Louis, MO), and 0.5% Through Path Plus (On-Chip Biotechnologies, Tokyo, Japan) in phosphate-buffered saline (PBS, Invitrogen)} to obtain a final concentration of 10^2^ cells/100 μL. From this suspension, tumor cells were individually picked up using a micropipette under an inverted microscope, and subsequently added to the 4 mL healthy blood sample. These spiked samples were then processed immediately via immunomagnetic enrichment as described below.

### Immunomagnetic enrichment and sample staining procedures

Immunomagnetic enrichment and sample staining of cells were described previously [30]. Briefly, samples were negatively enriched using Dynabeads coated with anti-CD45 monoclonal antibody (Invitrogen) to remove white blood cells, followed by fixation and labeling with the fluorescein isothiocyanate (FITC)-conjugated anti-CK mAb CK3-6H5 (1:25 dilution; Miltenyi Biotec, Bergisch-Gladbach, Germany), the PE-conjugated anti-vimentin mAb D21H3 (1:50 dilution; Cell Signaling Technology, Danvers, MA), and the Alexa Fluor 700-conjugated anti-CD45 mAb F10-89-4 (1:20 dilution; AbD Serotec, Oxford, UK). Samples were incubated overnight at 4°C in the dark, followed by stained with 1 μg/mL Hoechst 33342 (Sigma-Aldrich, St. Louis, MO) for 10 min at RT in the dark.

### On-chip Sort cell sorter

**Figure 1:**
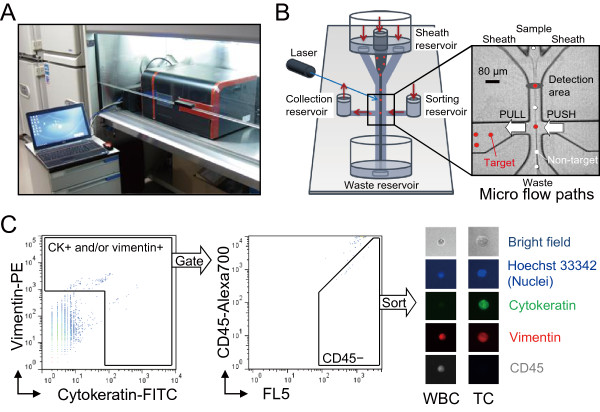
**Cell sorter instrument and gating mechanism for tumor cells. (A)** Image of the On-chip Sort cell sorter. **(B)** Image of the disposable microfluidic chip used. The chip is self-contained with both inlet and outlet sample/sheath reservoirs, which ensures that no fluids come into contact with the device. The target cells are separated to the collection reservoir by this shift of flow alone. A magnified view of the micro flow paths is shown in the inset on the right. **(C)** Sorting gates for spiked tumor cells. Tumor cells gated as cytokeratin + and/or vimentin+/CD45- were sorted. Nuclei, cytokeratin, vimentin and CD45 staining was used to distinguish tumor cells (Tumor cell, TC; nuclei+/CK+ and/or vimentin+/CD45-) from the white blood cell population (White blood cell, WBC; nuclei+/CK-/vimentin+/CD45+).

The novel cell sorter used in this study, On-chip Sort (On-Chip Biotechnologies, Tokyo, Japan), is a bench-top size sorter that is compatible with operation in most biosafety cabinets (Figure 1A). As shown in Figure 1B, the disposable microfluidic chip contains all fluidic and optical paths within a single, closed system, which is intended to realize cross contamination-free, biosafety adherent, lossless whole volume sorting. This system therefore provides suitable conditions for the capture of CTCs in a clinical setting. The principle of sorting will be described elsewhere.

The On-chip Sort of this paper has two excitation lasers (blue; 473 nm, 10 mW and red; 640 nm, 30 mW) and four detection channels, FL2 (509–552 nm), FL3 (565–605 nm), FL4 (576-620 nm), FL5 (658–695 nm), and FL6 (>700 nm). Signals for FITC, PE, Alexa Fluor 647, and Alexa Fluor 700 were collected through these detection channels. Voltages of each light sensor module of each channel are optimized for CTC detection (FSC: gain Low, SSC: 0.25 V, FL2: 0.35 V, FL3: 0.33 V, FL4:0.35 V, FL5: 0.48 V, and FL6: 0.35 V). On-chip Sort software version 24.1 (On-Chip Biotechnologies) was used for signal acquisition. Data analysis was performed using FlowJo software v7.6.5 (Tree Star, Inc, Ashland, OR).

### Enumeration and sorting procedures

Enumeration and sorting of cells were performed by On-chip Sort according to the manufacturer’s instructions. Briefly, the flow path was pre-washed with the 1 × Through Path Plus (On-Chip Biotechnologies) and the On-chip sample buffer (1 × Through Path Plus with 1.5% polyvinylpyrrolidone, On-Chip Biotechnologies). Stained samples were dissolved in 25 μL to 100 μL of On-chip sample buffer and then a flow rate was up to 150 events/sec (about 1 μL/min: 1.8 kPa in the On-chip Sort setting). Total events were approximately 1 × 10^5^ to 10^6^ events. The sorting time required for all the samples was approximately 30 to 120 minutes depending on the final sample volume.

The sorted cells gated into the cytokeratin and/or vimentin positive and CD45 negative channels were collected into the collection reservoir, and then observed under a fluorescence microscope (Biorevo BZ-9000: Keyence, Osaka, Japan) to confirm that the cells were nucleated (nuclear stain-positive), cytokeratin and/or vimentin positive and CD45 negative. All steps were carried out at room temperature.

### Whole genome amplification

Sorted cells were transferred from the collection reservoir to a 200 μL polymerase chain reaction (PCR) tube and rinsed the collection reservoir with sheath solution twice. After centrification (600 x g for 10 min), the supernatant was carefully aspirated to leave ~1 μL, which comprised the starting volume of the whole genome amplification (WGA) procedure. WGA was performed using the *Ampli*1 WGA kit (Silicon Biosystems, Bologna, Italy) following the manufacturer’s protocol. The AMPure XP PCR Purification Kit (Beckman Coulter, Beverly, MA) was used to clean up the amplified DNA, and DNA concentrations were determined using a NanoDrop spectrophotometer (NanoDrop Technologies, Waltham, MA, USA). Quality control checks of the WGA product were performed using the *Ampli*1 QC Kit (Silicon Biosystems). Only samples positive for four PCR products were considered to contain successfully amplified genomic material suitable for mutation analysis. Amplified DNA product of 2, 20 or 250 ng was subjected to mutation analysis using quantitative real-time-PCR (qPCR) amplification, pyrosequencing, or deep sequencing, respectively.

### Pyrosequencing

The amplification primers for mutations in *EGFR*, *KRAS*, and *BRAF* are described in Additional file 1: Table S1. Pyrosequencing PCR was performed following the manufacturer’s instructions.

### Deep sequencing using the TruSeq Amplicon Cancer Panel

A total of 48 genes frequently mutated in cancer according to the COSMIC database (Catalogue Of Somatic Mutations In Cancer), were sequenced using a TruSeq Amplicon Cancer Panel (TSACP; Illumina, San Diego, CA) following the manufacturer’s instructions. Variant call analysis was performed with Amplicon Viewer (Illumina). Coverage information was obtained using CLC genomics Workbench 6.0 (CLC Bio, Aarhus, Denmark).

### Mutation analysis of lung tumor cells enriched with the CellSearch profile kit

To compare the cell capture performance of the On-chip Sort platform versus the CellSearch platform (Veridex LLC), nine tubes (three regular 5 mL blood collection tubes containing EDTA) of blood were collected from a healthy volunteer. H1975, A549 or H1755 tumor cells were spiked into the 5 mL of blood to a final concentration of 10 cells/mL. Two blood collection tubes (total of 10 mL blood) were delivered to an independent medical laboratory (Genetic Lab, Sapporo, Japan). There, tumor cell capture was performed using the CellSearch profile kit (Veridex LLC) or the On-chip Sort in parallel concurrently. Captured samples using the CellSearch profile kit were stored in a CellSave Preservative Tube (Veridex LLC) and sent back to our laboratory. After a single wash with T-buffer, samples were stained as described above. Captured samples using the On-chip Sort were stored at 4°C until the initiation of WGA in parallel with returned CellSearch samples. Both samples were subjected to WGA concurrently, followed by mutation analysis.

### Gene copy number analysis for *EGFR*

qPCR amplification of the *EGFR* was performed on the StepOnePlus Real-time PCR system (Applied Biosystems, Foster City, CA) using SYBR Premix Ex Taq II (Tli RNase H Plus; Takara Bio, Shiga, Japan). The amplification primers used are described in Additional file 1: Table S1.

### Immunoblot analysis and immunofluorescence staining

Immunoblot analysis was as described previously [33]. Briefly, the cultured tumor cells were harvested and lysed in lysis buffer (50 mM Tris-HCI, pH 7.4, 50 mM NaCI, 1% Nonidet P-40, 2 mM EDTA, 10 mM NaF, 2 mM sodium orthovanadate and protease inhibitor cocktail). Whole cell lysate was electrophoresed on a 12% SDS-PAGE gel, transferred to nitrocellulose membrane (Bio-Rad Laboratories Inc., Hercules, CA) and immunoblotted with the a the phospho-EGFR (Tyr1068, D7A5; Cell Signaling), the EGFR (D38B1; Cell Signaling), or α-tubulin (YL1/2; Millipore, Temecula, CA). The intensity of the bands was quantified with ImageJ (Wayne Rasband, NIH, MD).

The cultured tumor cells were harvested and fixed. After washing with T-buffer once, the cell pellet was dissolved in a staining solution containing the PE-conjugated anti-CD326 (EpCAM) mAb 9C4 (1:25 dilution, BioLegend, San Diego, CA) or Alexa Fluor 647-conjugated anti-EGFR mAb D38B1 (Cell Signaling Technology). Samples were incubated overnight at 4°C in the dark. Unbound antibodies were removed via washing with 2 mL of T-buffer followed by centrifugation. Flow cytometry was performed using the On-chip Sort. Data analysis was performed using FlowJo software v7.6.5.

### Statistical analysis

Prism software (GraphPad Software, Inc., La Jolla, CA) was used for statistical analyses. Statistical significance of difference was determined using the unpaired Student’s *t*-test.

## Results

### Assay development for CTC detection and capture after negative depletion enrichment

To capture CTCs in whole blood, we used a novel cell sorter, On-chip Sort (Figure 1). Discrimination of CTCs from the bulk of the blood cells was achieved by negative enrichment using anti-human CD45 microbeads [30]. A typical example for the full gating strategy is shown in Figure 1C. Gating of the CTCs by On-chip Sort was carried out using the CK-FITC staining vs. the vimentin-PE staining density plot (Figure 1C, left). The lower limit of the gate that discriminates CTC signals from WBCs autofluorescence as well as from debris was determined by several runs of non-spike experiments using healthy donor control bloods (Additional file 2: Figure S1). The CK+ and/or vimentin+ events were then subjected to CD45 negative gating to distinguish tumor cells from WBCs and/or debris. Tumor cell events that appeared in the CD45-Alexa Fluor 700 vs. FL5 density plot (FL5 is a detection channel adjacent to that for Alexa Fluor 700) were easily distinguished from the WBCs and debris population (Figure 1C, middle).

Immunofluorescence staining of the On-chip Sort sorted cells identified these cells as nuclei, CK and/or vimentin-positive and CD45-negative under the fluorescent microscope, confirming these cells to be tumor cells. Tumor cells were easily distinguished from WBC, which were CD45-positive and CK-negative (Figure 1C, right). Five healthy donor control samples were processed with above settings (Additional file 2: Figure S1). On average these samples have 1.2 ± 1.3 events in the CTC gate. However no sorted tumor cells (CK+ and/or vimentin+, CD45- cells) were observed.

### Evaluation of captured tumor cells from spiked blood samples

**Figure 2:**
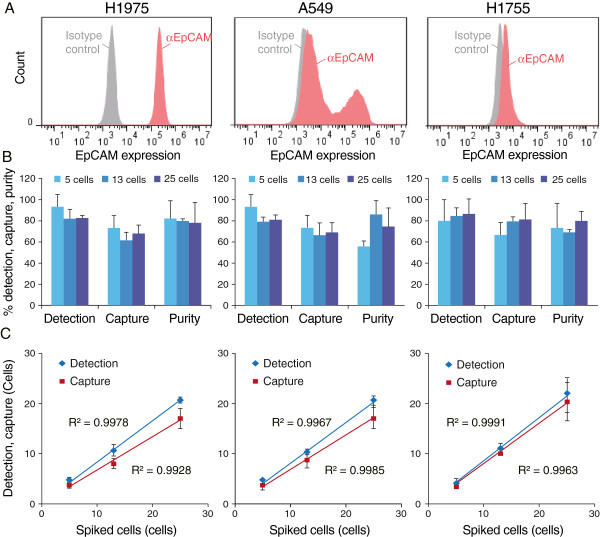
**Performance of On-chip Sort in a spike-in experiment. (A)** Histograms of EpCAM expression in H1975, A549, and H1755 non-small cell lung tumor cell lines. Fluorescence histograms of the isotype control (gray) and of the EpCAM antibody (red). **(B)** The mean percent detection, capture and purity of 5, 13, and 25 tumor cells isolated following spiking into 4 mL of normal blood (*n* = 3). The number of detected tumor cells was calculated using Flowjo software. The number of captured tumor cells was counted as the number of cells found in the collection reservoir. Purity was calculated as the number of captured tumor cells divided by the number of captured tumor cells plus white blood cells counted in the collection reservoir. **(C)** Linearity of tumor cell detection (blue lines) and capture (red lines) in spiking experiments. Error bars represent S.D. of the mean. Data shown here are representative of two independent experiments for each assay.

To assess the performance of our method, low numbers (i.e., 5, 13, or 25 cells) of various non-small cell lung tumor H1975, A549 or H1755 cells expressing varying levels of EpCAM were spiked into 4 mL of normal blood, and processed according to our protocol for tumor cell isolation with the On-chip Sort system. As shown in Figure 2A, H1975 cells displayed high EpCAM expression, whereas A549 cells exhibited partial EpCAM expression. H1755 cells did not appear to express any EpCAM.

Results of the sorting experiments are summarized in Figure 2B and in Additional file 3: Table S2. Overall, the mean percentages of cells detected into the gate as shown in Figure 1C were 86.9% ± 8.6%, 84.7% ± 9.3%, and 83.4% ± 12.4% for H1975, A549, and H1755 cells, respectively (*n* = 9). These detection rates are comparable to those obtained in the previous report using FISHMAN-R [30]. Captured cells into collecting reservoir were confirmed to be tumor cells using microscopy of cell morphology and positive CK and vimentin fluorescent labeling, while contaminating cells were identified by cell morphology and positive CD45 staining (Additional file 4: Figure S2). Overall mean percentages of cells captured were 68.5% ± 9.2%, 69.8% ± 9.9%, and 74.5% ± 12.7% for H1975, A549, and H1755 cells, respectively. Observation of CD45-positive cells under the fluorescent microscope yielded a purity of captured tumor cells after cell isolation with On-chip Sort of 78.4% ± 13.9%, 69.8% ± 18.5% and 70.4% ± 12.4% for H1975, A549, and H1755 cells, respectively.

Regression analysis of the number of detected tumor cells versus the number of expected tumor cells produced a correlation coefficient (R^2^) of 0.9978, 0.9967 and 0.9965 for H1975, A549, and H1755 cells, respectively (Figure 2C, blue line). The number of captured tumor cells was also highly linear, with a correlation coefficient of R^2^ = 0.9928, 0.9985 or 0.9963 in H1975, A549, or H1755 cells, respectively (Figure 2C, red line).

### Validation of mutation detection methods

Prior to performing mutation analysis on tumor cells captured with On-chip Sort, we tested whether WGA products are usable for sequencing by pyrosequencing and deep sequencing methods. The H1975 human lung tumor cell line harboring known mutations in the *EGFR* was used. Two single cells and two groups of ten cells each were analyzed for the presence of two different mutations in the *EGFR*; using both pyrosequencing and deep sequencing subsequent to the WGA procedure. Both mutations were reliably detected by pyrosequencing even in single cells, as well as in both unamplified and amplified H1975 genomic DNA carried out as a positive control (Table 1). These *EGFR* mutations were not detected in any of the amplified WBC samples carried out as a negative control (Table 1).

**Table 1:** Mutation analysis of single or small groups of tumor cells

**Cells**	**Template**	**EGFR mutation**	**Var. Freq. by Pyro.**	**Var. Freq. by MiSeq**		**Coverage min. = 10 × (212 amplicons)**
				**Var. Freq.**	**Total read**	
H1975	Unamplified gDNA	L858R (2573T>G)	75%	78.2%	13,070	100.0%
		T790M (2369C>T)	74%	79.5%	11,044	
H1975	Amplified gDNA	L858R	73%	65.9%	372	96.7%
		T790M	72%	75.2%	10,013	
H1975	10 cells	L858R	71%	88.8%	4,479	92.0%
		T790M	79%	84.1%	27,539	
H1975	10 cells	L858R	73%	90.1%	601	93.5%
		T790M	77%	80.9%	35,070	
H1975	1 cell	L858R	68%	77.1%	109	90.6%
		T790M	79%	87.0%	31,312	
H1975	1 cell	L858R	69%	78.2%	368	80.2%
		T790M	61%	63.8%	27,341	
WBC from healthy donor	10 cells	L858R	2%	N/A	3,596	81.4%
		T790M	0%	N/A	18,497	
WBC from healthy donor	1 cell	L858R	3%	N/A	56	88.7%
		T790M	0%	N/A	256	

Footnote: Tumor cells or normal WBCs were fixed and stained followed by *Ampli*1 WGA. WGA products were sequenced using a pyrosequencer or the Illumina MiSeq sequencer. Variant frequencies of two different mutations in the *EGFR* and the coverage distribution of WGA products using the TSACP are shown. Pyro.,psyrosequence Var. Freq., variant frequency. Coverage min., coverage minimum.

Amplicon libraries were generated using the TSACP followed by deep sequencing with an Illumina MiSeq sequencer. Significant single nucleotide variants (occurring in > 1% of DNA in the sample) were found in both the small groups of cells as well as in the single cells with satisfactory sequence coverage depth (Table 1). Similarly, A549 and H1755 human lung tumor cell lines harboring known mutations in the *KRAS* and *BRAF* were also reliably called with sufficient variant frequency when using small numbers of cells as well as in single cells (Additional file 5: Table S3).

Analytical sensitivities of pyrosequencing and deep sequencing were analyzed by titration studies using normal leukocyte and *EGFR* mutant H1975 cells. One or ten mutant cells were mixed with wild-type cells (normal leukocytes) in dilutions of 20, 5, and 1% of mutant cells. All cell mixtures were subjected to WGA, followed by sequencing with pyrosequencing and deep sequencing. Results are shown in Additional file 6: Figure S3. Even at mutant cell dilutions of 1%, the deep sequencing method was capable of detecting mutations in *EGFR* (Additional file 6: Figure S3B), while pyrosequencing had a lower detection limit of 10% (Additional file 6: Figure S3A).

### Mutation analysis of captured tumor cells

To enable the mutation analysis of CTCs isolated with On-chip Sort, the sorted lung tumor cell samples shown in Figure 2 were analyzed for the presence of specific mutations in each cell line using pyrosequencing and deep sequencing. Variant frequencies were compared with those of both unamplified and amplified H1975 genomic DNA samples. Results of the mutation analysis of the cells sorted for WGA and the purity of these samples are summarized in Additional file 3: Table S2. All expected mutations were reliably detected in all sorted samples with sufficient coverage, with none detected in the amplified WBC samples. These results suggest that even where tumor cells were present at concentrations as low as five cells per 4 mL of blood, they could be successfully isolated using On-chip Sort, amplified genome DNA by WGA, and profiled by deep sequencing with sufficient depth.

We further evaluated whether On-chip Sort was capable of capturing very low number of tumor cells in blood. 4 mL blood samples containing one or two H1975 cells were processed with On-chip Sort in 6 independent tests (Additional file 7: Table S4). The results demonstrated a sensitivity threshold for On-chip Sort platform detecting close to one tumor cell per 4 mL of blood. In addition, tumor cells were not detected from healthy donor blood containing no tumor cells. Therefore, CTCs could be detected and isolated form patients who have ≥1 CTCs per 4 mL of blood by the On-chip Sort platform and they could be genotyped utilizing isolated CTCs.

We also found that EpCAM/CK double-negative Hs578T cells spiked into healthy blood were successfully isolated with On-chip Sort and profiled by mutation analysis (Additional file 8: Figure S4); the resulting mutation profiles showed the expected genomic mutation in p53 known to be present in Hs578T. These results strongly suggest that our CTC capture assay is advantageous for capturing and characterizing both EpCAM-positive and EpCAM-negative tumor cells.

### Comparative analysis of On-chip sort versus the CellSearch profile kit

**Figure 3:**
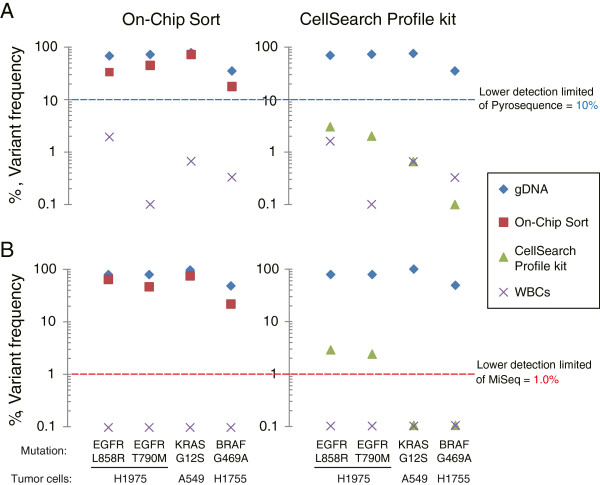
**Head-to-head comparison of On-chip Sort with the CellSearch profile kit.** Three tumor cell lines (H1975, A549 and H1755) expressing varying levels of EpCAM were spiked into blood from a healthy donor (10 cells/mL). The samples were divided into three and then processed using our On-chip Sort protocol as well as the CellSearch profile kit in parallel. Captured tumor cells were subjected to whole genome amplification followed by mutation detection with both pyrosequencing and deep sequencing to detect specific mutations in each tumor cell. Variant frequencies detected in the controls (gDNA and WBCs from a healthy donor) and experimental samples using pyrosequencing **(A)** or deep sequencing **(B)** are graphically represented. The line represents the lower limit of detection of each method (10% for pyrosequencing and 1% for deep sequencing). Samples with 0% variant frequency were assigned a value of 0.1 for plotting purposes. Data shown here are representative of two independent experiments for each assay.

We next performed a comparative analysis of On-chip Sort versus the CellSearch profile kit in terms of mutation detection of tumor cells spiked into blood samples. Cells isolated using On-chip Sort or the CellSearch profile kit were processed by WGA followed by mutation analysis with pyrosequencing or deep sequencing. In On-chip Sort-isolated samples, specific mutations in each tumor cell line were reliably detected by both pyrosequencing and deep sequencing (Figure 3, left). In CellSearch profile kit-isolated samples however, specific mutations in H1975 cells expressing high EpCAM levels were detected by deep sequencing only and not by pyrosequencing (Figure 3, right). In both experiments, genomic DNA was successfully amplified according to *Ampli*1 end-point PCR criteria in all of the samples (Additional file 9: Figure S5). Details of the mutation analysis are shown in Additional file 10: Table S5. These data suggest that the On-chip Sort assay provides superior sensitivity compared with the CellSearch profile kit for subsequent mutation detection.

### Assessment of EGFR expression and copy number amplification in captured tumor cells

**Figure 4:**
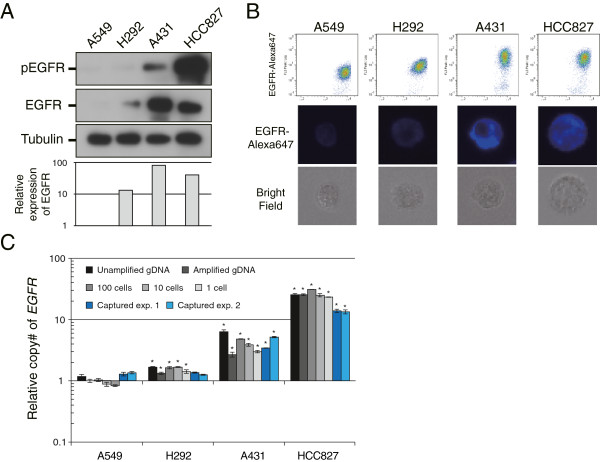
**EGFR protein expression and *EGFR *amplification in tumor cells captured by On-ship Sort. (A, B)** EGFR protein expression detected by western blot **(A)**, correlated with EGFR protein expression captured by On-chip Sort **(B)**, for A549 low EGFR expression and no EGFR amplification, H292 (moderate EGFR expression and amplification rate), A431 (strong EGFR expression and amplification rate), and HCC827 (strong EGFR expression and large amplification rate) cell lines. **(C)** Comparison of *EGFR* amplification rates determined by qPCR with gDNA (black bar), and when using*Ampli*1 WGA products (gray bars) including WGA of captured tumor cells (blue bars).

Owing to the multichannel detection capability of the system, EGFR expression levels on single CTCs are measurable and semi-quantifiable using an anti-EGFR antibody in the FL5 channel of On-chip Sort. We first performed experiments to demonstrate that EGFR immunostaining on On-chip Sort could be correlated to EGFR expression and gene amplification status as determined by western blot analysis and qPCR using WGA products from various cell lines. To distinguish different levels of EGFR protein expression and *EGFR* amplification, we used the EGFR-over-expressing and/or EGFR-amplified cell lines H292, A431, and HCC827 (Figure 4A, C). Very low-level EGFR-expressing A549 cells with a single *EGFR* copy served as a negative control (Figure 4A, C).A549 cells were either weakly positive or negative, whereas H292 cells also revealed moderate EGFR expression (Figure 4B). In A431 and HCC827 cells, strong intensities of EGFR specific immunofluorescence were observed (Figure 4B). These EGFR expression levels correlated with those determined by immunoblot analysis (Figure 4A). CTCs with moderate to strong intensities of EGFR-specific immunofluorescence were assumed to be EGFR-positive (H292, A431, and HC827), whereas CTCs with negative or only weak intensities of EGFR-specific immunofluorescence were considered to be EGFR-negative (A549) (Figure 4B).

EGFR expression levels determined using On-chip Sort were consistent with the mean gene amplifications determined by qPCR carried out on DNA extracts of the corresponding cell lines (Figure 4C, black bars; A549, 1.18-fold; H292, 1.69-fold; A431, 6.32-fold; HCC827, 25.37-fold). In line with these results, the analysis of *Ampli*1 WGA products of genomic DNA (1 ng) and of small numbers of cells including single cells, by *EGFR* qPCR, revealed comparable mean values in the respective cell lines (Figure 4C, grey bars). The mean gene amplifications of pure tumor cell samples (Figure 4C, grey bars) and of those isolated by On-chip Sort (Figure 4C, blue bars) were compared, and found to be reasonably similar to that observed in the cell lines with strong expression levels of EGFR (A431 and HC827; Figure 4C), whereas no significant amplification was detected in H292, which expresses moderate levels of EGFR (Figure 4C). The results of these sorting experiments are summarized in Additional file 11: Table S6.

## Discussion

In this study, we have described a new approach for the capture of rare tumor cells from immunomagnetically pre-enriched blood samples. We provided proof-of-principle demonstrating the feasibility of this approach by using it to capture between one and ten tumor cells from spiked blood samples for subsequent molecular characterization at the genomic level using deep sequencing.

In addition, we investigated the applicability of using WGA products obtained from single CTCs with the *Ampli*1 WGA kit. This WGA kit is the only commercially available kit compatible with analysis of a single fixed cell. Fixation is a crucial step for the staining of cytokeratin and vimentin with fluorescent probes, and cytokeratin positivity is included to gold standard criteria for CTC detection. The WGA/sequencing procedure described in this study was shown to be serviceable for the detection of a broad range of somatic mutations in 48 cancer-related genes in small numbers of cells, as well as in single cells for the first time. A coverage depth of 10-fold was achieved even for single cells at >90% of the nucleotide positions. Next-generation sequencing-based diagnostics may therefore hold the potential to provide clinically relevant information using single CTCs.

Total CTC capture yield is important in genotyping as well as in other applications, such as prognostic and drug response measurements. The overall mean percentage of cells captured was 70.9% ± 10.6% (*n* = 27). The percentage of cell loss attributed to the negative enrichment procedure and sorting was about 20% and 10%, respectively. The estimated capture limit of the number of tumor cells captured by our system was 1.46 ± 0.22 CTCs/4 mL blood. The cut-off number of CTC events detected by the On-chip Sort method was 3 events/4 mL blood (Additional file 2: Figure S1), suggesting that the CTC capture efficiency of our system to detect mutation might be sufficient for low CTC cohort numbers. In fact, we were able to detect specific mutations from blood samples containing one tumor cell.

In a low CTC cohort (<15 CTCs/7.5 mL), the rate of successful subsequent WGA was reported to be 23.5% (*n* = 34) after isolation using a conventional cell sorter [20]. Cell loss during sample handling is a critical issue in CTC isolation when using CellSearch [21,28]. Unlike conventional cell sorters and CellSearch, the On-chip Sort system employs a lossless whole volume sorting approach, and as a result, displays 100% successful subsequent WGA as well as mutation detection of spiked blood samples as low as five tumor cells in 4 mL blood (*n* = 9).

Purity of isolated CTCs is also crucial for obtaining high sensitivity in mutation detection. The On-chip Sort system isolated tumor cells at a purity of >60%, which is sufficient for mutation detection using pyrosequencing methods that have a sensitivity of approximately 10%. When directly comparing mutation detection sensitivity of our system with that of the CellSearch system and profile kit, our system was observed to be significantly more sensitive at low CTC numbers (<10 CTCs/mL). Detection of variant frequency of *EGFR* mutations in high EpCAM-expressing H1975 cells after our sorting procedure was approximately 68%, which was sufficient to call the mutations. However, in the same samples using the CellSearch system, variant frequency was approximately 3%, which is insufficient for unambiguous variant identification. These results suggest that our system displays superior purification efficiency for the detection of mutations using the relatively low-sensitivity downstream pyrosequencing method.

The use of EMT markers, e.g., vimentin, facilitated the capture of EpCAM/CK double-negative tumor cell in peripheral blood. The loss of EpCAM and/or CK in tumor cells has been reported previously [34-39]. This loss of epithelial cell properties is related to Epithelial-to-Mesenchymal Transition (EMT). Our multicolor cell sorting system allows us to capture a CTC-positive marker and an EMT-related marker in parallel, suggesting that a population of CTCs that has been missed by current platforms might be able to capture and characterize using our system. However, vimentin is also expressed on mesenchymal stromal cells which normally circulate [40]. Criteria for vimentin+ CTCs must be carefully defined and evaluated in future clinical studies. We also consider using other EMT-related makers such as N-cadherin or twist in addition to vimentin staining to detect and capture CTCs which show mesenchymal phenotype [34,38]. Our multicolor cell sorting system equipped with multilaser has the potential to capture CTCs multiple EMT markers. In addition to CTC markers, nuclear staining positivity is regarded one of the golden standard criteria to detect CTCs. It is important to incorporate nuclear positivity in sorting gates to classify the events as CTCs or not in the case of clinical samples. On-chip Sort can be equipped with violet laser for detection of DAPI staining. Further improvement of equipment is needed to be a robust diagnostic tool for cancer patients.

While the use of *Ampli*1 technology has previously been reported for PCR-based mutation analysis on single cells [21,22], qPCR to determine copy number of single cells using the *Ampli*1 kit has not yet been evaluated. In our study, the mean gene amplification rates within various tumor cells determined by qPCR on DNA extracts were similar to those determined with *Ampli*1 WGA products when using both DNA extracts as well as small numbers of cells, including single cells. The amplification of the *EGFR* could also be observed in captured samples, suggesting our system might be capable for detecting gene amplification on CTCs. Such analyses may assist in predictive biomarker studies in cases where expression levels of therapeutic targets may be predictive of therapeutic activity. For example, High EGFR expression of tumor cells was shown to predict the benefit of anti-EGFR therapy such as cetuximab [41].

Evaluation of the clinical feasibility of phenotypic analysis in captured CTCs by evaluating target gene expression is still ongoing. The DETECT III trial assesses the use of anti-HER2 treatments in HER2-negative breast cancer patients selected on the basis of CTC detection/characterization [42], http://www.detect-studien.de. Results of this trial will give an insight into the relevance of CTCs in cancer treatment strategies. Our system has clear advantages over conventional systems when carrying out longitudinal analyses of CTC dynamics in terms of protein expression as well as of mutation status.

## Conclusions

We have provided the first report on the performance of the On-chip Sort system, a novel bench-top cell sorter that allows the capture of low numbers of cells from human blood samples. We have described the analytical characteristics of the system and provided proof-of-principle showing its feasibility in the capture and molecular characterization of low numbers of tumor cells in blood. Ongoing improvement of CTC enrichment processes from whole blood and integration of single-cell technologies may help to establish CTCs as a pivotal diagnostic tool for cancer patients towards enabling better-personalized therapies. The data shown in this study imply the potential of the On-chip Sort cell capture system and further evaluation with clinical samples should be conducted.

## Abbreviations

CTCs: Circulating tumor cells; EpCAM: Epithelial cell adhesion molecule; qPCR: Quantitative polymerase chain reaction; WBCs: White blood cells; FITC: Fluorescein isothiocyanate; CK: Cytokeratin; PE: Phycoerythrin; WGA: Whole genome amplification; TSACP: TruSeq Amplicon Cancer Panel.

## Competing interests

KT represents the manufacturer of On-chip Sort.

## Authors’ contributions

MW and YK conceived and designed the experiments. MW performed the experiments except the deep sequencing. MS performed the deep sequencing. MW analyzed the data and conducted the statistical analysis. MS analyzed the coverage data of deep sequencing. MW, MS, TS, KT, TT, NY, FK, and YK contributed reagents, materials, and analysis tools. MW and YK wrote the paper. All authors read and approved the final manuscript.

## Supplementary Material

Additional file 1: Table S1 Primers used for pyrosequencing and quantitative PCR (qPCR). *1: 5′ ends of the amplification primers were biotinylated. Click here for file

Additional file 2: Figure S1 Detection and sorting data of healthy donor control samples. A typical example of healthy control samples analyzed with On-chip Sort. On average five healthy donor control samples have 1.2 ± 1.3 events (*n* = 5) in the CTC gate (CK+ and/or vimentin+/CD45-), but no tumor cells were observe in the collecting reservoir. Click here for file

Additional file 3: Table S2 Details of mutation analysis of captured cells. This table provides details of DNA from captured tumor cells shown in Figure 2, which was amplified using *Ampli*1 WGA followed by mutation detection with both a pyrosequencer and an Illumina MiSeq sequencer. Variant frequencies of two different *EGFR* mutations and single *KRAS* and *BRAF* mutations, as well as coverage distribution of WGA products in the TruSeq Amplicon Cancer Panel are shown. Var. Freq., variant frequency. Coverage min., coverage minimum. Click here for file

Additional file 4: Figure S2 Gallery of H1975 cells captured by On-chip Sort in the collection reservoir. Captured cells are shown with binding to fluorescently-labeled antibodies targeting cytokeratin, vimentin, and CD45. The images allowed for identification of tumor cells (arrow) and hematologic cells (arrowheads). Click here for file

Additional file 5: Table S3 Mutation analysis of single or small groups of A549 and H1755 cells. Tumor cells were fixed and stained followed by *Ampli*1 WGA. WGA products were sequenced using a pyrosequencer or an Illumina MiSeq sequencer. Variant frequency of *KRAS* or *BRAF* mutations and WGA coverage distribution in the TruSeq Amplicon Cancer Panel are shown. Var. Freq., variant frequency. Coverage min., coverage minimum. Click here for file

Additional file 6: Figure S3 Analytical sensitivity of mutation detection. Dilutions of *EGFR* mutant H1975 cells spiked into healthy donor WBCs were analyzed by both pyrosequencing and deep sequencing for detection of T790M and L858R mutations. Variant frequencies of *EGFR* mutations detected by the pyrosequencer (A) or MiSeq sequencer (B) are graphically represented. The horizontal axis shows the expected fraction of mutant *EGFR* cells. The vertical axis shows the observed percentage of variant frequency. The variant frequencies of the T790M mutation (diamonds) and of the L858R mutation (squares) are indicated. Blue marks indicate dilutions of single H1975 cell into WBC samples and green marks indicate dilutions of ten H1975 cells into WBC samples. The line represents the lower limit of detection of the method (10% for pyrosequencing and 1% for deep sequencing). Data shown here are representative of two independent experiments for each assay. Click here for file

Additional file 7: Table S4 Evaluation of sensitivity of On-chip Sort platform for mutation detection. One or two cultured H1975 cells were individually picked up using a micropipette under an inverted microscope, spiked into 4 mL aliquots of healthy donor blood, and the resulting blood samples were processed using the On-chip Sort platform in 6 separate tests. Captured samples were analyzed for the presence of specific mutations in each cell line using pyrosequencing. Click here for file

Additional file 8: Figure S4 Capture and mutation profiling of CK-/EpCAM - breast cancer cells. (A) Histograms of CK, EpCAM, and vimentin expression in Hs578T cells. Fluorescence histograms of the isotype control (gray) and of the EpCAM antibody (red). (B) CTC gates of spiked Hs578T cells and gallery of Hs578T cells captured by On-chip Sort. The images allowed for identification of Hs578T cells (arrow). (C) Details of sorting results and mutation analysis using deep sequencing. DNA from captured Hs578T cells was amplified using *Ampli*1 WGA followed by mutation detection with an Illumina MiSeq sequencer. Variant frequencies of *p53* mutation and coverage distribution of WGA products in the TSACP are shown. Var. Freq., variant frequency. Coverage min., coverage minimum. Click here for file

Additional file 9: Figure S5 Composite gel images of *Ampli*1 QC end-point PCR products. Genomic DNA of experimental samples was considered to be successfully amplified if all four of the control genomic DNA sequences were detected. No amplification product was obtained in either of the negative control samples (NTC and Buffer). All of the captured samples obtained using either On-chip Sort or the CellSearch Profile kit passed the *Ampli*1 amplification check. NTC, no template control; gDNA, 1 ng of H1975 gDNA as a positive control for *Ampli*1 QC; Buffer, negative control for WGA. Click here for file

Additional file 10: Table S5 Details of mutation analysis of captured tumor cells using On-chip Sort or the CellSearch profile kit. This table provides details of the captured sample in Figure 3 that were subjected to mutation analysis. DNA from captured tumor cells were amplified using *Ampli*1 WGA followed by mutation detection using both the pyrosequencer and the Illumina MiSeq sequencer. Variant frequencies of two different *EGFR* mutations, single *KRAS* and *BRAF* mutations, and coverage distribution of WGA products in the TruSeq Amplicon Cancer Panel are shown. Var. Freq., variant frequency. Coverage min., coverage minimum. Click here for file

Additional file 11: Table S6 Capture efficiencies and purity of tumor cells spiked into 4 mL of normal blood. This table provides details of the captured samples show in Figure 4 that were subjected to copy number analysis. The number of captured tumor cells was counted as the number of tumor cells found in the collection reservoir. Purity was calculated as the number of captured tumor cells divided by the number of captured tumor cells plus the number of white blood cells counted in the collection reservoir (*n* = 2). Click here for file
