# Supplementary figures and images for: A novel flow cytometry-based cell capture platform for the detection, capture and molecular characterization of rare tumor cells in blood
Source: J Transl Med. 2014 May 23;12:143. doi: 10.1186/1479-5876-12-143 (PMC4053587; doi:10.1186/1479-5876-12-143)

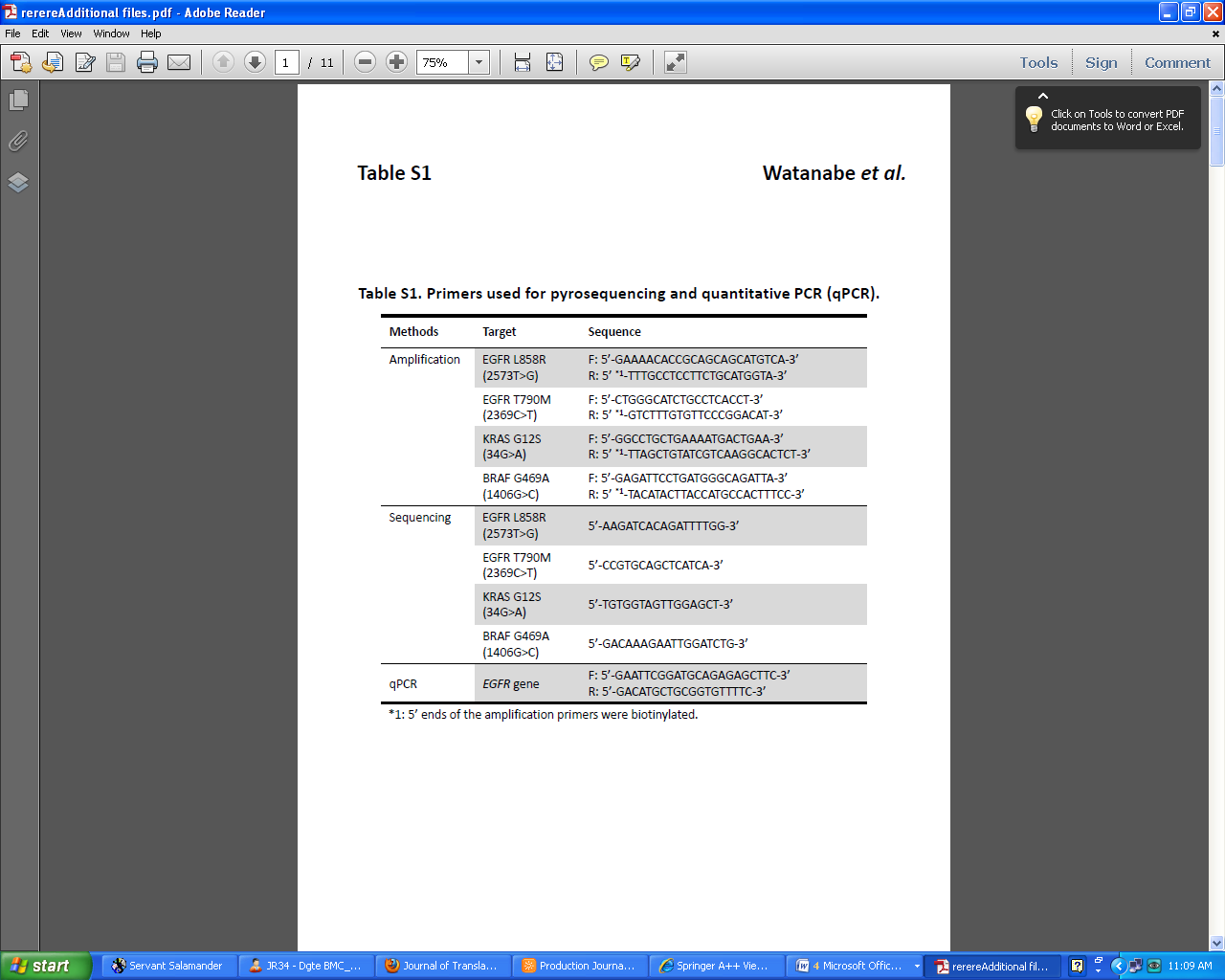

Supplement: Additional file 1: Table S1 — Primers used for pyrosequencing and quantitative PCR (qPCR). *1: 5′ ends of the amplification primers were biotinylated. [file 1479-5876-12-143-S1.doc]

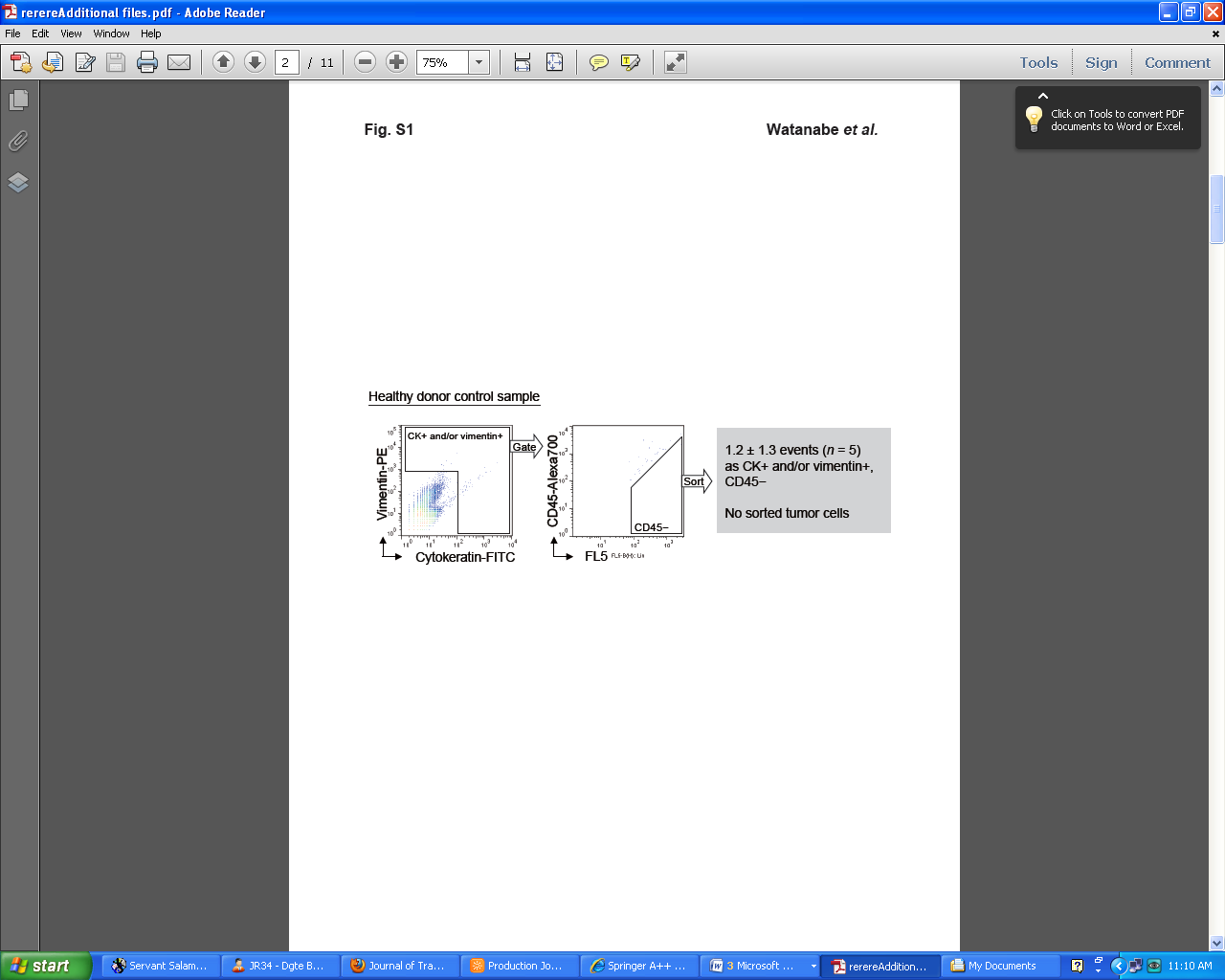

Supplement: Additional file 2: Figure S1 — Detection and sorting data of healthy donor control samples. A typical example of healthy control samples analyzed with On-chip Sort. On average five healthy donor control samples have 1.2 ± 1.3 events (n = 5) in the CTC gate (CK+ and/or vimentin+/CD45-), but no tumor cells were observe in the collecting reservoir. [file 1479-5876-12-143-S2.doc]

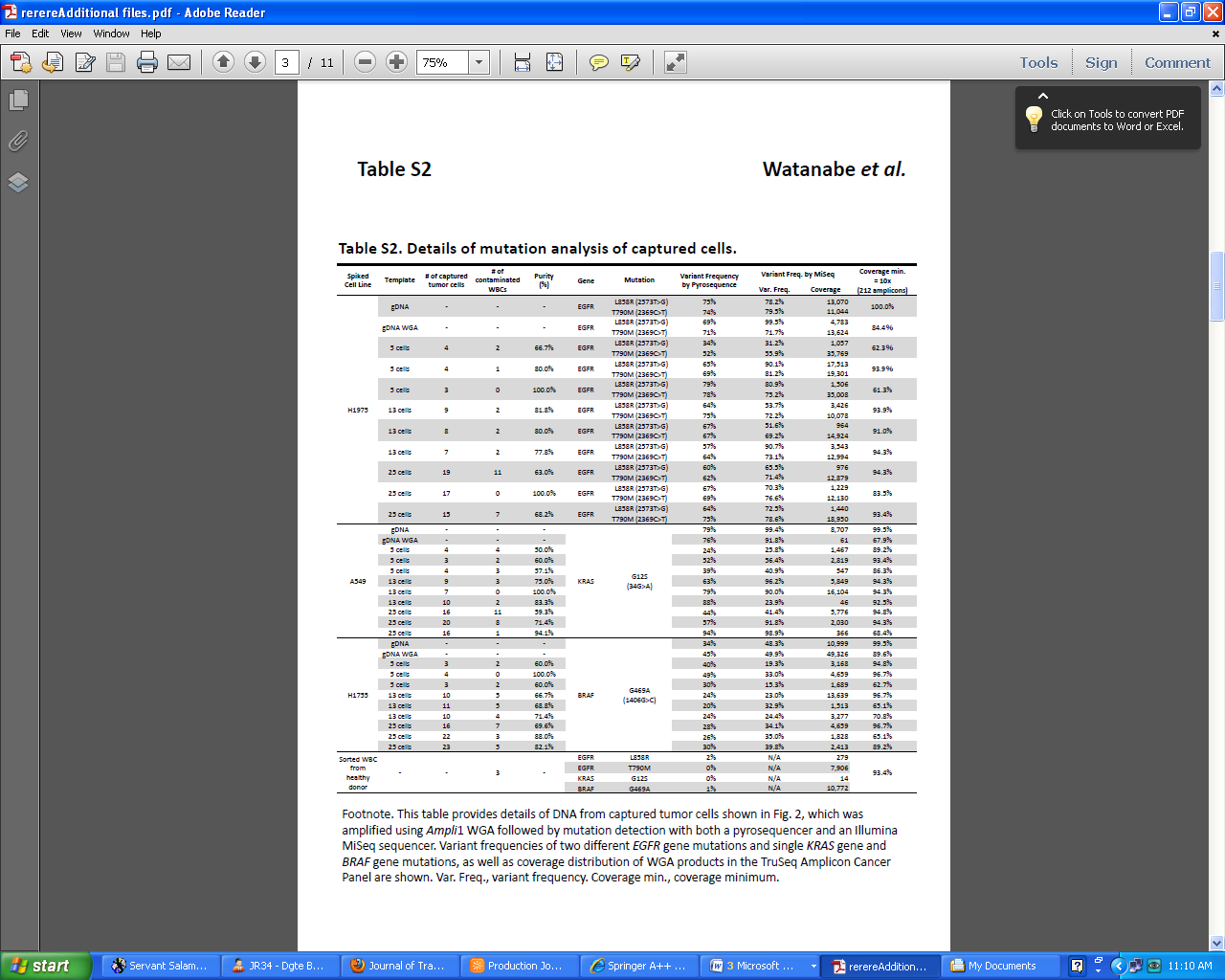

Supplement: Additional file 3: Table S2 — Details of mutation analysis of captured cells. This table provides details of DNA from captured tumor cells shown in Figure 2, which was amplified using Ampli1 WGA followed by mutation detection with both a pyrosequencer and an Illumina MiSeq sequencer. Variant frequencies of two different EGFR mutations and single KRAS and BRAF mutations, as well as coverage distribution of WGA products in the TruSeq Amplicon Cancer Panel are shown. Var. Freq., variant frequency. Coverage min., coverage minimum. [file 1479-5876-12-143-S3.doc]

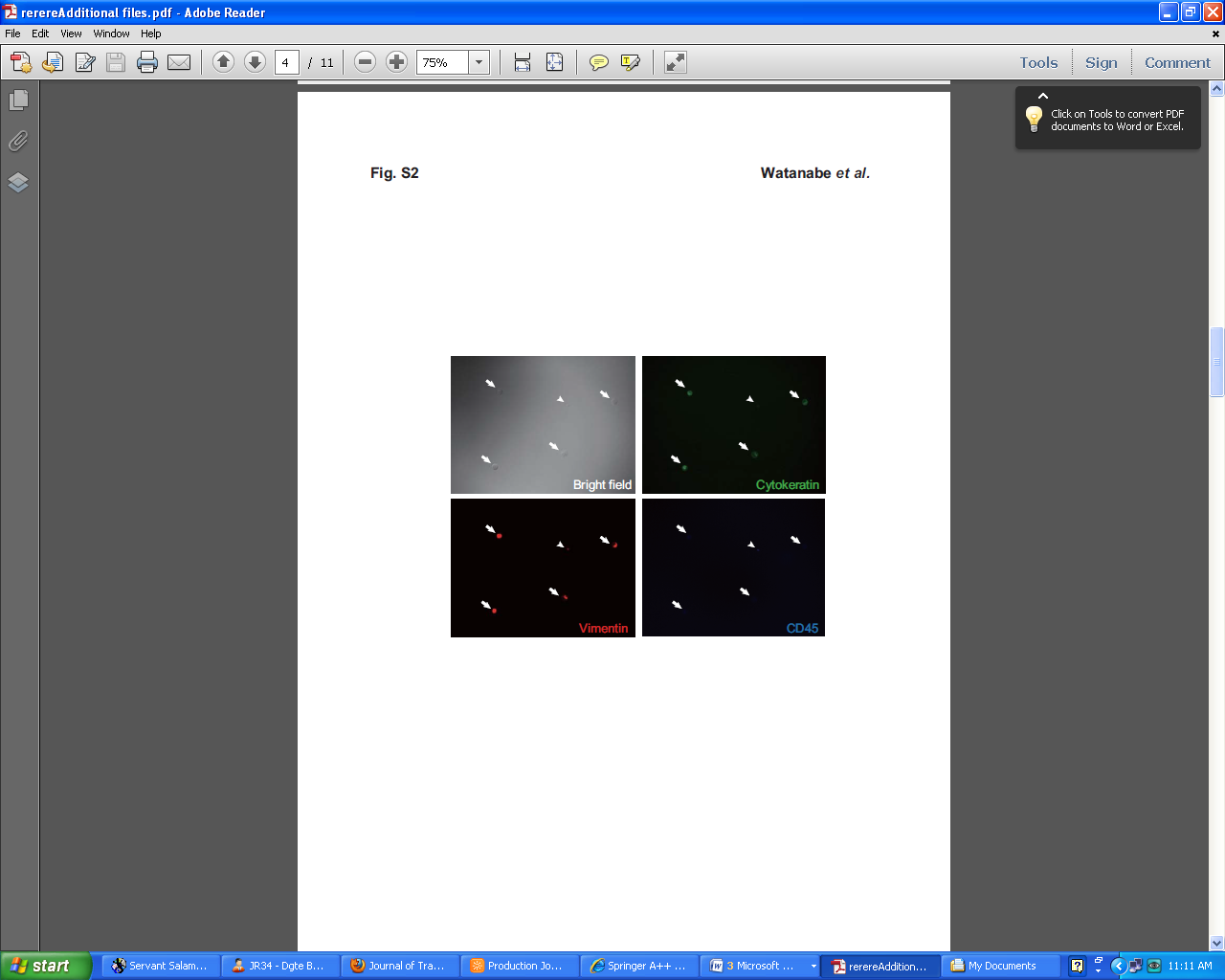

Supplement: Additional file 4: Figure S2 — Gallery of H1975 cells captured by On-chip Sort in the collection reservoir. Captured cells are shown with binding to fluorescently-labeled antibodies targeting cytokeratin, vimentin, and CD45. The images allowed for identification of tumor cells (arrow) and hematologic cells (arrowheads). [file 1479-5876-12-143-S4.doc]

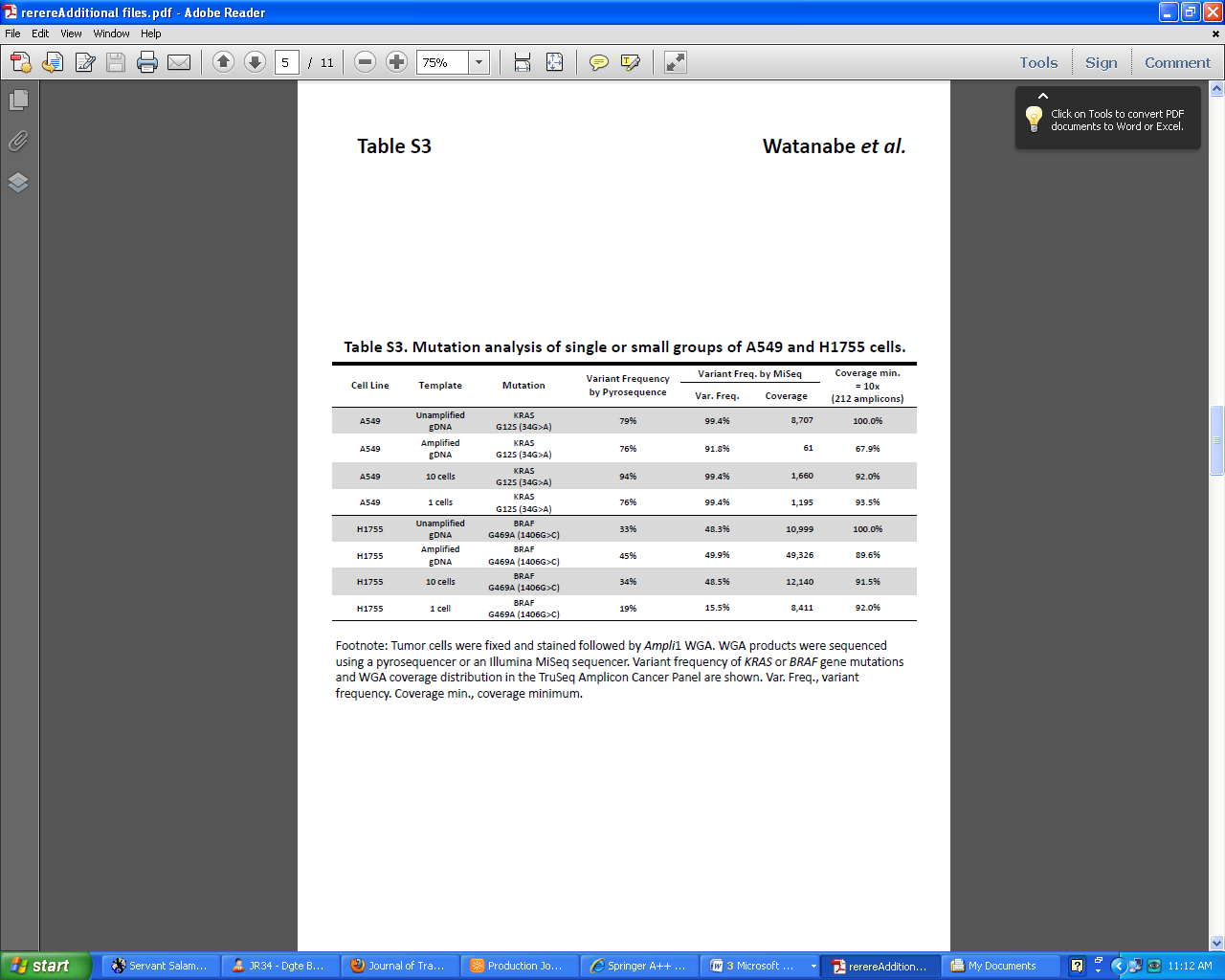

Supplement: Additional file 5: Table S3 — Mutation analysis of single or small groups of A549 and H1755 cells. Tumor cells were fixed and stained followed by Ampli1 WGA. WGA products were sequenced using a pyrosequencer or an Illumina MiSeq sequencer. Variant frequency of KRAS or BRAF mutations and WGA coverage distribution in the TruSeq Amplicon Cancer Panel are shown. Var. Freq., variant frequency. Coverage min., coverage minimum. [file 1479-5876-12-143-S5.doc]

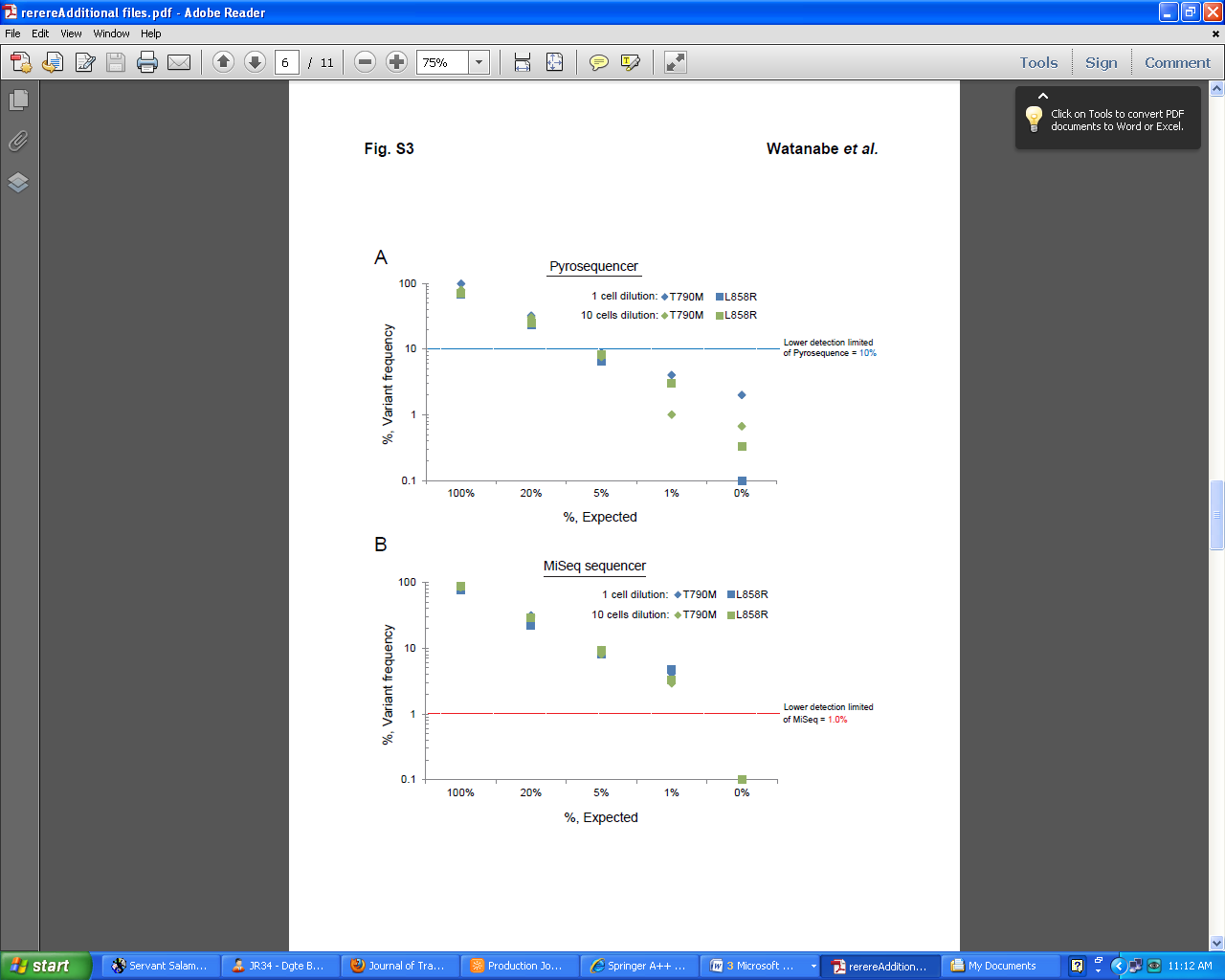

Supplement: Additional file 6: Figure S3 — Analytical sensitivity of mutation detection. Dilutions of EGFR mutant H1975 cells spiked into healthy donor WBCs were analyzed by both pyrosequencing and deep sequencing for detection of T790M and L858R mutations. Variant frequencies of EGFR mutations detected by the pyrosequencer (A) or MiSeq sequencer (B) are graphically represented. The horizontal axis shows the expected fraction of mutant EGFR cells. The vertical axis shows the observed percentage of variant frequency. The variant frequencies of the T790M mutation (diamonds) and of the L858R mutation (squares) are indicated. Blue marks indicate dilutions of single H1975 cell into WBC samples and green marks indicate dilutions of ten H1975 cells into WBC samples. The line represents the lower limit of detection of the method (10% for pyrosequencing and 1% for deep sequencing). Data shown here are representative of two independent experiments for each assay. [file 1479-5876-12-143-S6.doc]

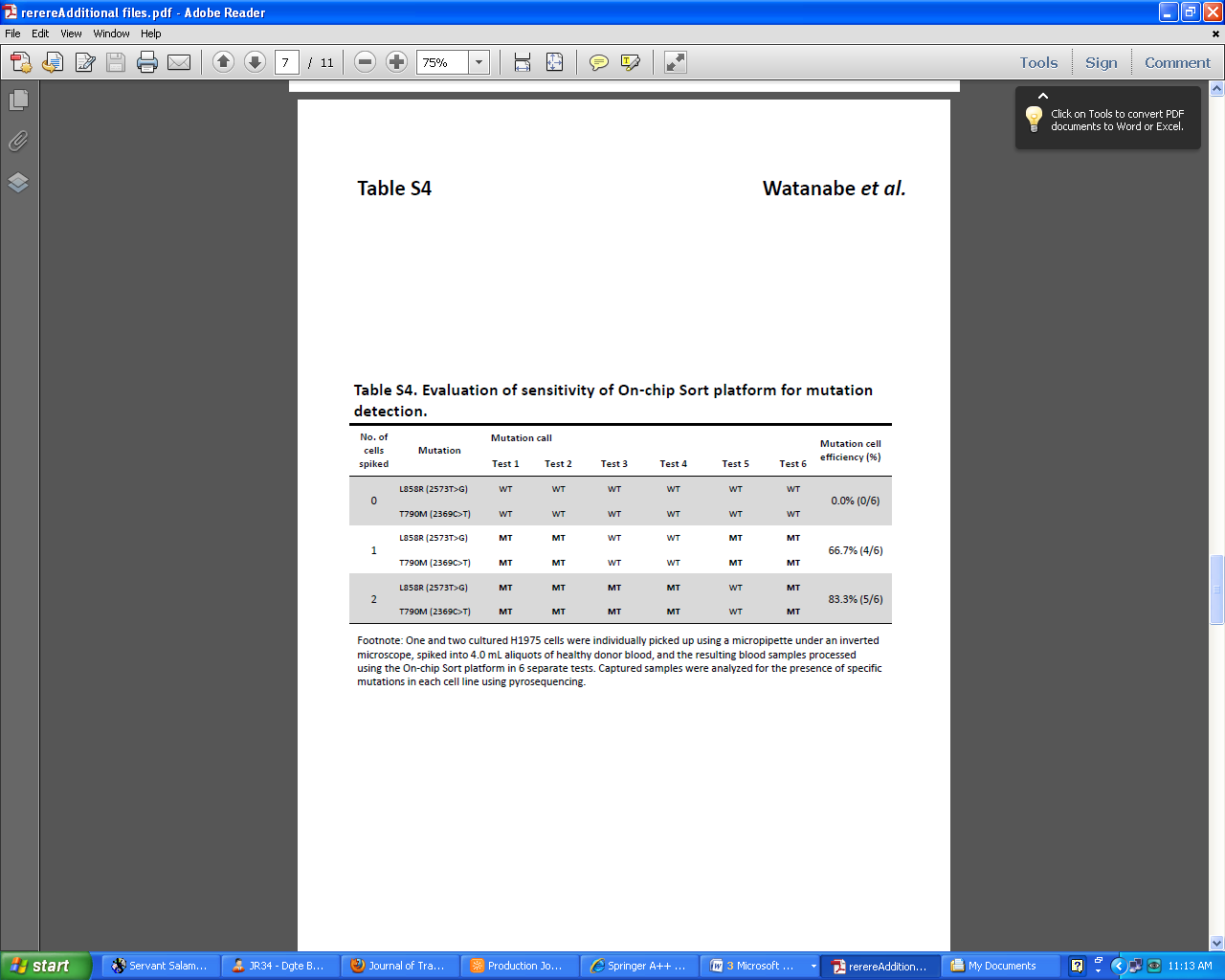

Supplement: Additional file 7: Table S4 — Evaluation of sensitivity of On-chip Sort platform for mutation detection. One or two cultured H1975 cells were individually picked up using a micropipette under an inverted microscope, spiked into 4 mL aliquots of healthy donor blood, and the resulting blood samples were processed using the On-chip Sort platform in 6 separate tests. Captured samples were analyzed for the presence of specific mutations in each cell line using pyrosequencing. [file 1479-5876-12-143-S7.doc]

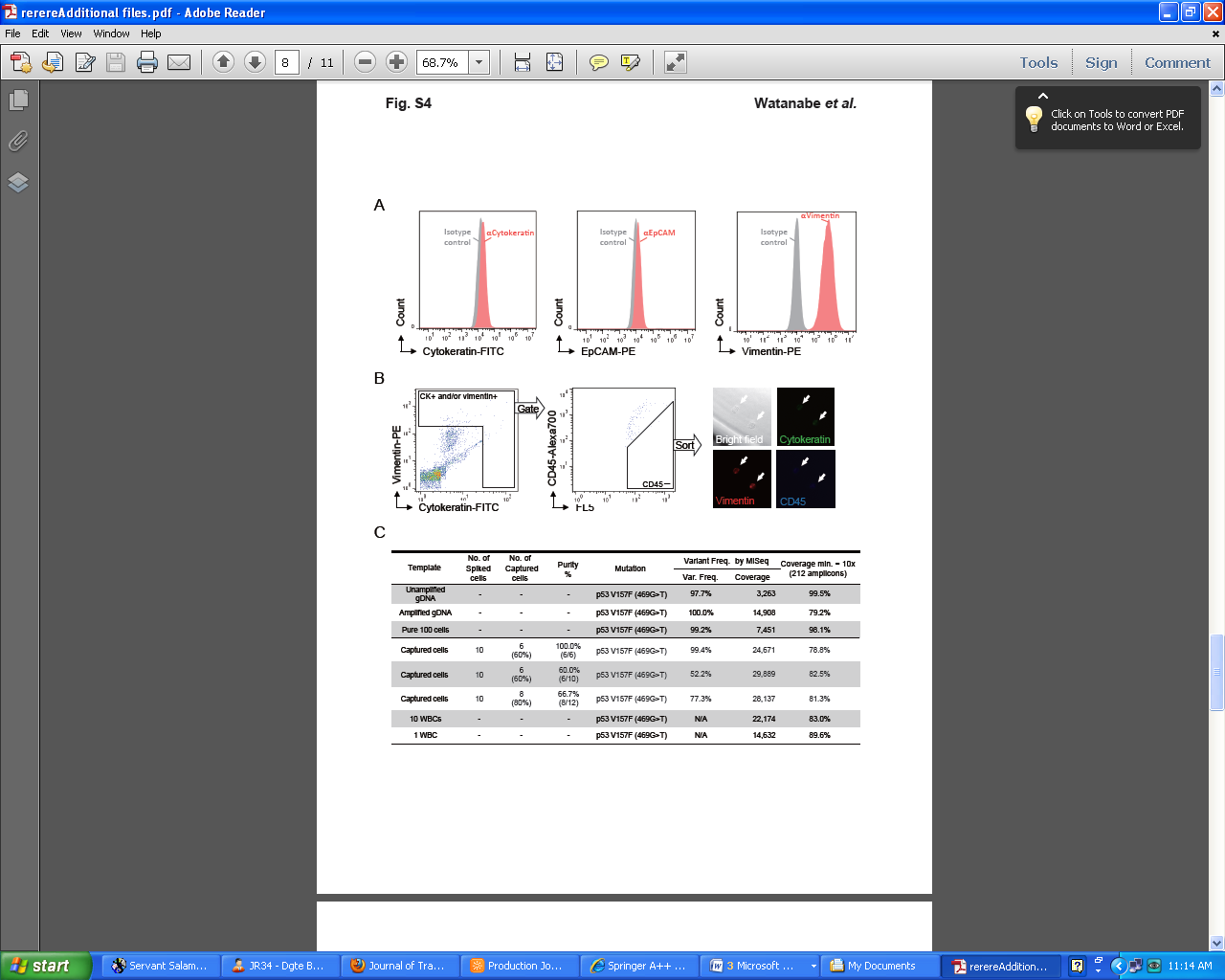

Supplement: Additional file 8: Figure S4 — Capture and mutation profiling of CK-/EpCAM - breast cancer cells. (A) Histograms of CK, EpCAM, and vimentin expression in Hs578T cells. Fluorescence histograms of the isotype control (gray) and of the EpCAM antibody (red). (B) CTC gates of spiked Hs578T cells and gallery of Hs578T cells captured by On-chip Sort. The images allowed for identification of Hs578T cells (arrow). (C) Details of sorting results and mutation analysis using deep sequencing. DNA from captured Hs578T cells was amplified using Ampli1 WGA followed by mutation detection with an Illumina MiSeq sequencer. Variant frequencies of p53 mutation and coverage distribution of WGA products in the TSACP are shown. Var. Freq., variant frequency. Coverage min., coverage minimum. [file 1479-5876-12-143-S8.doc]

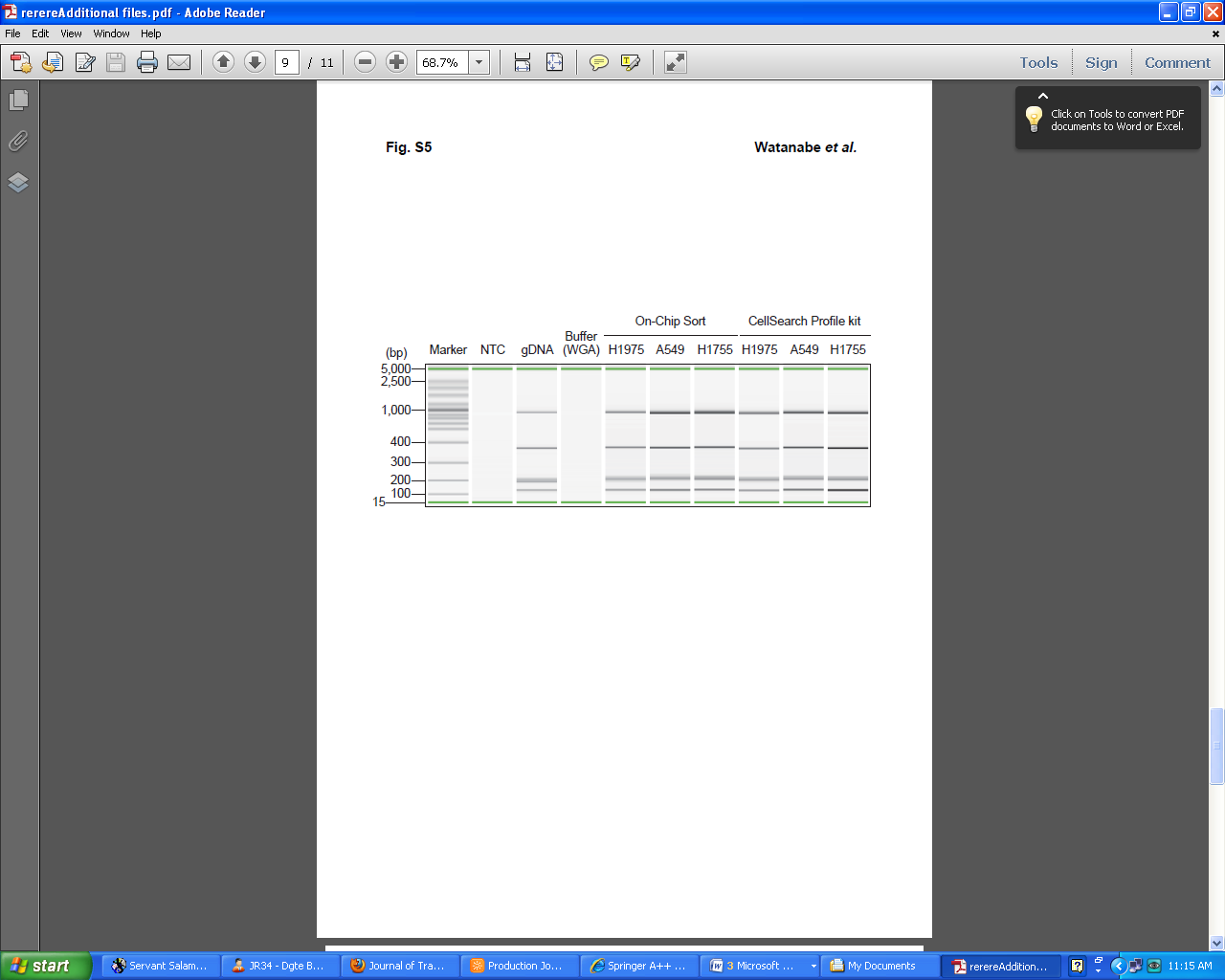

Supplement: Additional file 9: Figure S5 — Composite gel images of Ampli1 QC end-point PCR products. Genomic DNA of experimental samples was considered to be successfully amplified if all four of the control genomic DNA sequences were detected. No amplification product was obtained in either of the negative control samples (NTC and Buffer). All of the captured samples obtained using either On-chip Sort or the CellSearch Profile kit passed the Ampli1 amplification check. NTC, no template control; gDNA, 1 ng of H1975 gDNA as a positive control for Ampli1 QC; Buffer, negative control for WGA. [file 1479-5876-12-143-S9.doc]

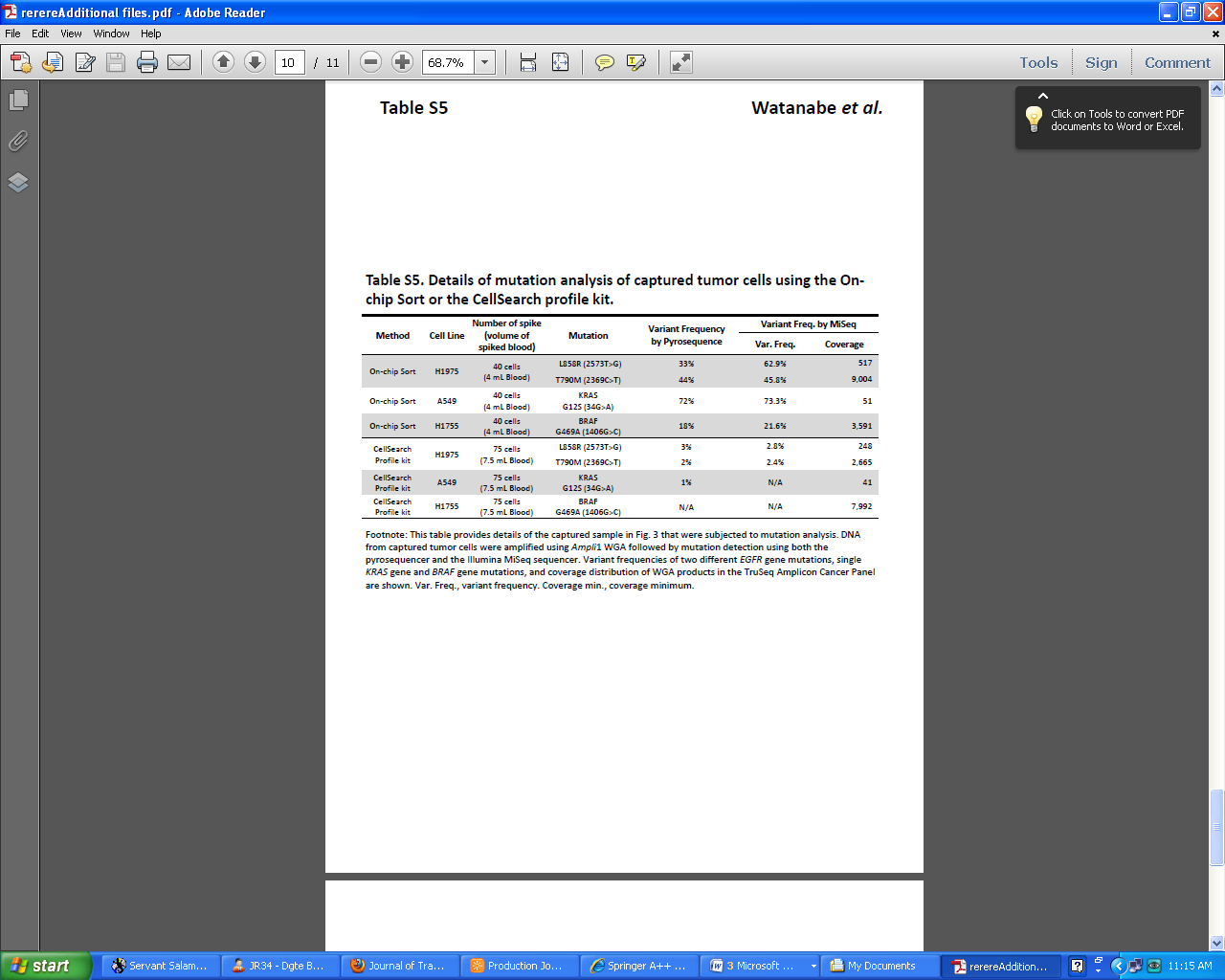

Supplement: Additional file 10: Table S5 — Details of mutation analysis of captured tumor cells using On-chip Sort or the CellSearch profile kit. This table provides details of the captured sample in Figure 3 that were subjected to mutation analysis. DNA from captured tumor cells were amplified using Ampli1 WGA followed by mutation detection using both the pyrosequencer and the Illumina MiSeq sequencer. Variant frequencies of two different EGFR mutations, single KRAS and BRAF mutations, and coverage distribution of WGA products in the TruSeq Amplicon Cancer Panel are shown. Var. Freq., variant frequency. Coverage min., coverage minimum. [file 1479-5876-12-143-S10.doc]

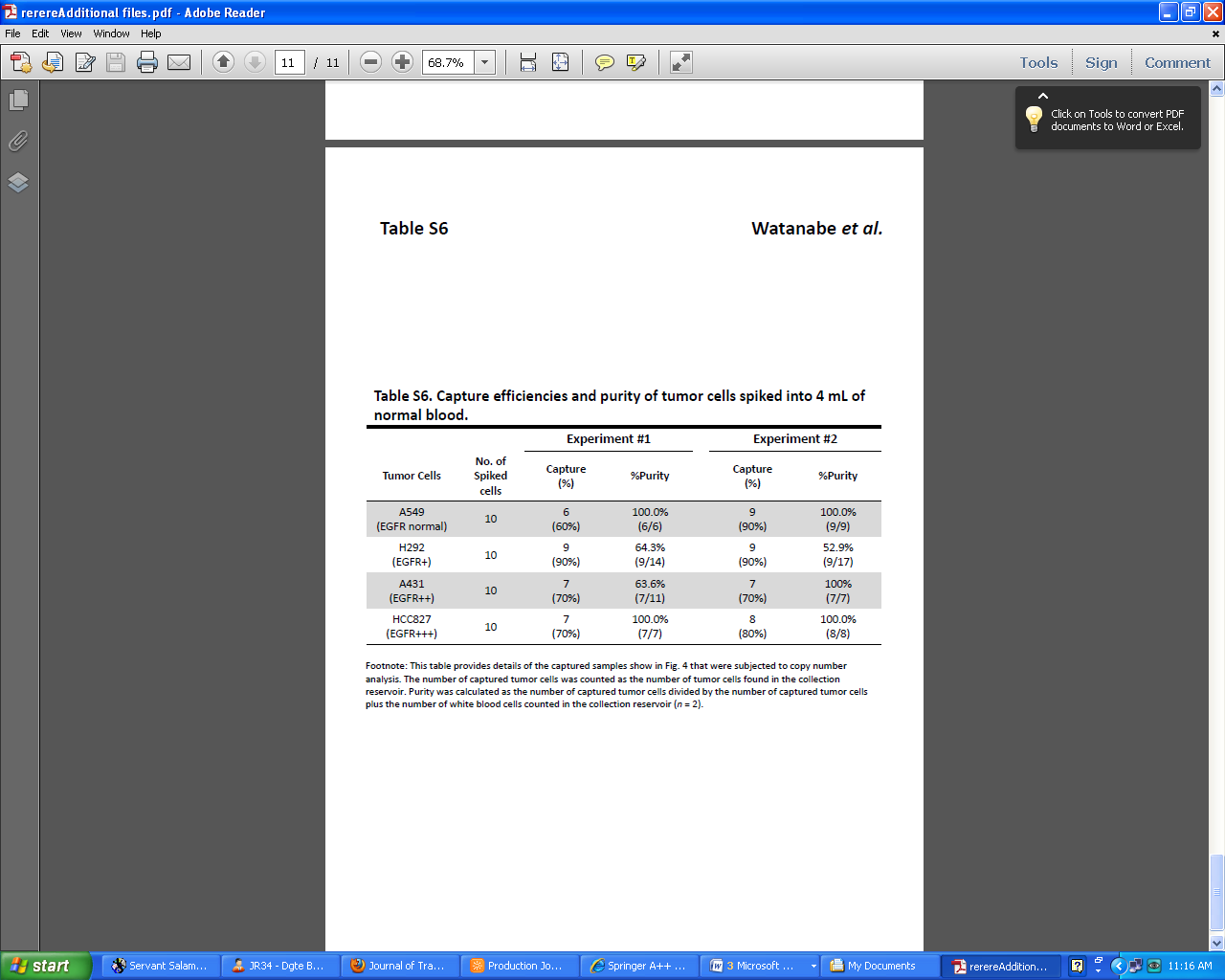

Supplement: Additional file 11: Table S6 — Capture efficiencies and purity of tumor cells spiked into 4 mL of normal blood. This table provides details of the captured samples show in Figure 4 that were subjected to copy number analysis. The number of captured tumor cells was counted as the number of tumor cells found in the collection reservoir. Purity was calculated as the number of captured tumor cells divided by the number of captured tumor cells plus the number of white blood cells counted in the collection reservoir (n = 2). [file 1479-5876-12-143-S11.doc]
